# A Dual-Transducer Approach for High-Resolution and High-Precision Shear Wave Elasticity Imaging

**DOI:** 10.3390/s25175532

**Published:** 2025-09-05

**Authors:** Jingfei Liu, Stanislav Y. Emelianov

**Affiliations:** 1 Department of Mechanical Engineering, Texas Tech University, Lubbock, TX 79409, USA; 2 School of Electrical and Computer Engineering, Georgia Institute of Technology, Atlanta, GA 30332, USA; stas@gatech.edu; 3 Wallace H. Coulter Department of Biomedical Engineering, Georgia Institute of Technology and Emory University School of Medicine, Atlanta, GA 30332, USA

**Keywords:** contrast-to-noise ratio, precision, shear wave elasticity imaging, signal-to-noise ratio, spatial resolution

## Abstract

Shear wave elasticity imaging, an ultrasound-based method for imaging tissue elasticity, has been widely accepted in both preclinical studies and clinical practices for diagnosing various diseases. Currently, shear wave elasticity imaging is primarily implemented using a single-transducer approach, in which the same ultrasound transducer is used for both generating and recording shear waves in target tissue. This technical implementation well served the need for imaging bulk tissues in various cases. However, the limited bandwidth of the ultrasound transducer is a great obstacle to extending the application of shear wave elasticity imaging to cases where higher spatial resolution and/or stronger tissue stimulation are needed. To address this challenge, we proposed a dual-transducer approach in which two ultrasound transducers perform shear wave generation and tracking, each optimized for its respective task. The feasibility of the proposed method is demonstrated and verified in a phantom study. In this pioneering work, the strength of the dual-transducer approach is shown by its performance in shear wave tracking at various frequencies. This performance is evaluated by four measures: signal-to-noise ratio, contrast-to-noise ratio, spatial resolution, and precision in quantitative measurement. The experimental results demonstrate the superior elasticity imaging capabilities of the dual-transducer approach compared to the conventional single-transducer approach, offering a reliable strategy for further development of this imaging method for specific applications.

## 1. Introduction

Shear wave elasticity imaging (SWEI), or shear wave elastography, is an ultrasound-based elasticity imaging method that can provide a quantitative characterization of tissue elasticity (shear modulus or Young’s modulus) in a two-dimensional field of view [1,2,3,4,5]. This method utilizes acoustic radiation force impulse (ARFI) to remotely generate shear waves within target tissues and employs ultrafast ultrasound imaging [6] to track the propagation of these shear waves. The speed of the recorded shear wave is then acquired locally and converted to tissue elasticity. To date, this technique has become a standard function of primary commercial ultrasound machines, such as LOGIQ E9/E10/S8/P9 by GE Healthcare, ACUSON S, Sequoia, and X700 by Siemens Healthineers, EPIQ and Affiniti by Pillips, and RS80A and RS85 by Samsung Medison. Clinically, SWEI has been established as an effective tool for diagnosing various diseases, including liver diseases [7,8,9,10], breast diseases [11,12,13], and thyroid diseases [14,15,16]. It has also been used in examining musculoskeletal organs such as tendons [17,18,19], ligaments [20,21,22], and muscles [23,24,25]. And its application is still expanding to image other organs, such as the kidney [26,27], prostate [28,29], and pancreas [30,31].

The current clinical implementation of SWEI is based on a single-transducer approach, in which an array transducer, either a linear array (e.g., GE ML6-15-D) or a convex array (e.g., GE C1-6-D), is used for both generating and tracking shear waves in tissues under test. Since the same transducer can accomplish both shear wave generation and tracking, this single-transducer approach has the great advantage of being convenient and simple in operation. However, in the attempt to apply SWEI to more challenging situations, such as evaluating the elasticity of small tissues or organs like lymph nodes or coronary arteries, especially in research environments working with small animals, this approach faces critical challenges.

One of these critical challenges is the lack of adequate spatial resolution. Currently, SWEI is primarily implemented at a relatively lower frequency range (not exceeding 10 MHz) in clinical applications. Although this frequency range can meet the requirements of many applications, it cannot provide the sufficient spatial resolution needed for imaging small tissues or organs. A natural solution for improving the spatial resolution and accuracy of SWEI is to adopt high-frequency transducers (whose center frequency is greater than 10 MHz) [32] despite alternative improvements using phase velocity estimation and a data-driven approach [33]. While high-frequency SWEI can provide high resolution in shear wave tracking, it poses another critical challenge: low effectiveness in generating shear waves. First, the piezoelectric elements of high-frequency transducers are not designed to sustain high-voltage and long-duration pulses that are required for producing ARFI [34]. Furthermore, even if ARFI is excited at high frequencies, it will have a lower penetration depth and a lower amplitude compared to low-frequency excitation, due to the lower excitation voltage allowed by high-frequency transducers and the higher attenuation of acoustic intensity at higher frequencies [35]. The low ARFI penetration depth prevents imaging deep tissue targets, and its low amplitude results in a low signal-to-noise ratio (SNR) of the generated shear wave, both of which make it ineffective for SWEI. To induce shear waves with higher intensity and/or at a deeper depth, low-frequency ultrasound is desired; while to achieve higher imaging resolution, high-frequency ultrasound is required. However, any ultrasound transducer has a limited bandwidth (which is typically indicated by its name), and the frequencies required for optimal shear wave generation and tracking may exceed this bandwidth. Therefore, the limited bandwidth of a single transducer makes it impossible to achieve effective shear wave generation and high-resolution shear wave tracking simultaneously for applications such as imaging small organs or tissue structures deep inside the human or animal body.

To address this challenge and avoid the tradeoff between spatial resolution and shear wave SNR when using the single-transducer approach for SWEI, a dual-transducer approach, in which two transducers of choice can implement shear wave generation and tracking separately, can be an effective solution. The purpose of assigning the tasks of generating and tracking shear waves to two transducers is to achieve optimized performance in each task by choosing a transducer with an appropriate frequency range and other specifications. The shear wave excitation transducer can be a single-element transducer or an array transducer, and it generally should have a low working frequency. The shear wave tracking transducer is usually an array transducer with a higher center frequency and a wider bandwidth.

In medical ultrasound, the dual-transducer idea has initially been adopted for ultrasound image-guided focused ultrasound therapy, which utilizes two transducers, one for imaging and the other for therapy [36,37]. In ultrasound elasticity imaging, this dual-transducer idea has been implemented in harmonic motion imaging, where a focused ultrasound transducer is used to generate oscillatory acoustic radiation force (harmonic waves) to excite the target tissue, while an imaging transducer is used to track the dynamic response of the tissue [38]. In elasticity imaging, this idea has recently also been used in cases where shear waves are generated using mechanical vibrators or actuators, while tracked using ultrasound transducers [39,40].

Despite the dual-transducer concept having been technically implemented in several cases of elasticity imaging research, it remains a relatively new idea to SWEI, and its performance has not been evaluated. Among the many factors affecting the output of SWEI, the performance of SWEI is mainly determined by two factors: the effectiveness of shear wave excitation and the quality of shear wave tracking. As a pioneer effort to assess the performance of dual-transducer SWEI, this work will focus on investigating the effects of shear wave tracking frequencies on the performance of SWEI. The performance of SWEI will be evaluated by four measures: SNR, contrast-to-noise ratio (CNR), spatial resolution, and precision in quantitative measurement. The experimental results demonstrate the superior effectiveness of high-frequency tracking over low-frequency tracking in SWEI by comparing the performance of SWEI at three different frequencies.

## 2. Materials and Methods

### 2.1. Phantom

The investigations were performed on four tissue-mimicking phantoms composed of 4% weight/volume (*w*/*v*) gelatin from porcine skin (Sigma-Aldrich, Co., St. Louis, MO, USA) and 0.2% *w*/*v* silica gel (Product Number 288500, Sigma-Aldrich, Co., St. Louis, MO, USA). One of the phantoms is homogeneous and was designed for evaluating the noise level of the imaging system. The other three phantoms each contain a cylindrical inclusion made of 10% *w*/*v* gelatin and 0.25% *w*/*v* silica gel, and they were designed to assess the performance of the proposed dual-transducer approach for SWEI at different imaging frequencies. The three inclusions are 4.3 mm, 3 mm, and 2.3 mm in diameter, respectively, and they are embedded at depths of 18 mm, 12 mm, and 10 mm from the phantom surface.

### 2.2. Experiment

**Figure 1:**
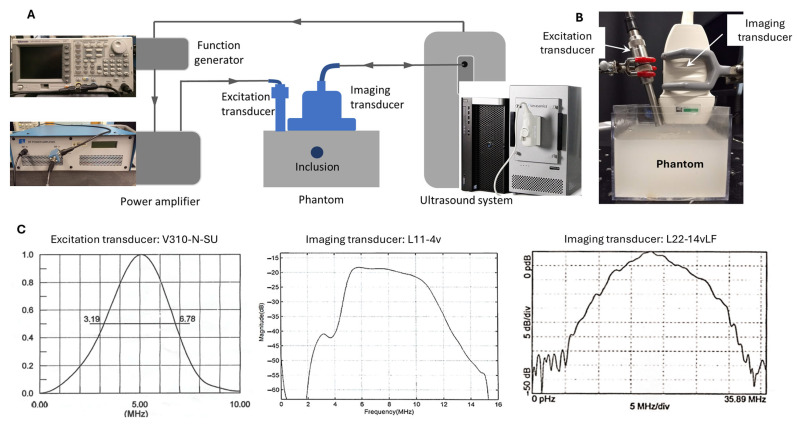
(**A**) Schematic of the phantom and experimental setup with the photographs of the key equipment. (**B**) The photograph of the layout of the phantom, the shear wave excitation, and imaging transducers. Shear waves are generated by a low-frequency single-element excitation transducer and recorded by a high-frequency linear array imaging transducer. (**C**) The frequency spectra of the shear wave excitation transducer and the two shear wave tracking transducers. All the spectra are provided by the manufacturers.

The experimental setup of this study is depicted in Figure 1. Unlike the conventional SWEI configuration, in which shear wave generation and tracking are performed by the same system (the single-transducer approach), the proposed dual-transducer approach executes shear wave generation and tracking in two separate subsystems. The shear wave generation subsystems consists of a function generator (AFG3022C, Tektronix Inc., Beaverton, OR 97077, USA), a power amplifier (E&I A150, Electronics and Innovation, LTD, Rochester, NY 14623, USA), and an excitation transducer (V310-N-SU, Olympus America Inc., Waltham, MA 02453, USA); its function is to generate an ARFI with enough power to excite shear waves in target tissues. The shear wave tracking subsystem consists of a programmable ultrasound research system (Vantage 128, Verasonics Inc., Redmond, WA, USA) and a linear array imaging transducer; its function is to record the propagating shear waves generated in the target tissues through ultrafast ultrasound imaging [6]. In operation, these two subsystems were synchronized by a trigger sent from the ultrasound system to the function generator.

For shear wave excitation, a 5 MHz focused single-element transducer was chosen to achieve deeper penetration (due to its low frequency and accordingly low attenuation) and a narrower excitation site (due to its focused nature). To reduce the distance between the excitation site and the imaging transducer, a pencil case style, which is only half the diameter of the typical case style, was chosen for the excitation transducer. To generate an ARFI signal that can effectively produce shear waves in the phantoms of this study, a sine-wave tone burst of 2000 cycles (i.e., 400 µs at 5 MHz) with a peak-to-peak voltage of 1.5 V was generated by the function generator and sent to the amplifier before being fed to the excitation transducer.

To study the effects of ultrasound imaging frequency on SWEI, the generated shear waves were tracked at three frequencies using two linear array transducers: 5 MHz and 10 MHz by L11-4v (Verasonics Inc., Redmond, WA, USA), and 15 MHz by L22-14vLF (Verasonics Inc., Redmond, WA, USA). The selection of these three shear wave tracking frequencies is based on the following facts: (i) the shear wave imaging frequency in clinical practice can be as low as 1–6 MHz [10], and (ii) the frequency in preclinical studies can be as high as 15–17.5 MHz [32,41]. So, the selected three frequencies can be very representative of the entire frequency range used in SWEI. To ensure a fair comparison can be made among these three frequencies, the same experimental parameters (in software settings, hardware settings, and physical configuration) were used during data acquisition, except for the imaging frequency. It should be noted that when acquiring data for evaluating and comparing the noise level of the imaging system at different frequencies, only the imaging subsystem functioned. Therefore, no shear wave was observed, and only system noise was recorded.

### 2.3. Signal Processing

**Figure 2:**
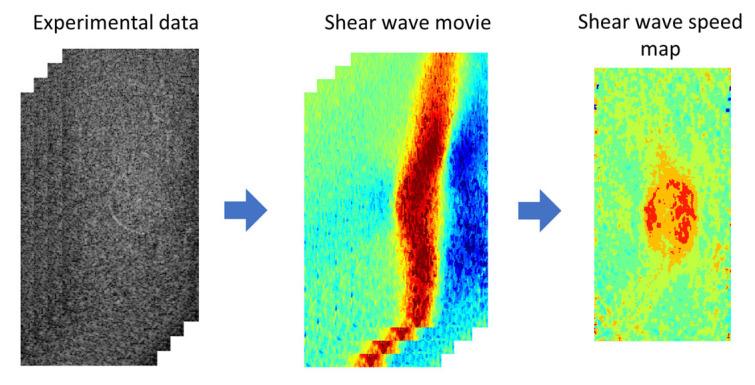
The signal processing workflow in shear wave elasticity imaging.

In SWEI, the process of signal processing (Figure 2) consists of two steps: (i) converting the experimental data to a shear wave movie and (ii) extracting a shear wave speed map from the shear wave movie. In this study, the experimental data are the in-phase quadrature (IQ) data acquired in ultrafast ultrasound imaging, which record the time-varying material displacement (usually invisible to the human eye) of the medium generated by ARFI excitation. In experiments, displacements of the target medium occurred in different directions of the three-dimensional space, but only the axial and lateral displacements were recorded in the acquired IQ data. Because the ARFI excitation was made in the axial direction of the imaging plane, the axial displacement is much larger in amplitude than the lateral displacement. Therefore, in the first step of signal processing, axial displacement was extracted from the acquired IQ data to represent the propagating shear waves. Then, by tracking the movement of the shear waves, a shear wave speed map was obtained for the entire imaging plane. All data processing was implemented offline using MATLAB (2021b, MathWorks Inc., Natick, MA, USA). To ensure a fair comparison, the same parameters were also used in processing data acquired at three different frequencies.

### 2.4. Noise Level Evaluation

Noise is an inherent characteristic of an imaging system, although it is not desired. In an output image, noise is a generic term that refers to any type of random fluctuation, which can significantly impact image quality, including the signal-to-noise ratio (SNR), contrast-to-noise ratio (CNR), and even spatial resolution [42]. Therefore, it is meaningful and necessary to evaluate the noise level of an imaging system to assist in the development and assessment of imaging methods.

The source and amount of noise depend on the imaging system (including both software and hardware) used and the signal processing methods employed. In this study, we will not investigate the exact sources of noise but rather evaluate the amount of noise to compare the performance of different imaging frequencies. As introduced in the subsection “Signal Processing”, in SWEI, data are stored in three forms: IQ data (acquired experimentally) for recording tissue motion, axial displacement for demonstrating shear wave, and shear wave speed for evaluating material elasticity. The noise originated from the experimental hardware and software of the imaging system, which is initially contained in the IQ data and carried on into the other two types of data. In comparison, the noise (including artifacts) originated from the signal processing and is only contained in the latter two types of data.

As mentioned in the subsections of “Phantom” and “Experiment”, a homogeneous gelatin phantom was made for noise evaluation, and it was imaged at three different frequencies without external excitation for shear wave generation. To evaluate the noise generated by the imaging system, the acquired IQ data were first converted to B-mode images, and a representative region of interest (ROI) of 10 mm by 10 mm was chosen in the imaging field of view (FOV) for computing the mean signal intensity and the associated standard deviation (STD). Here, the noise of the imaging system is defined as the STD of the ROI with the assumption that noise is distributed uniformly throughout the imaging FOV. To ensure a fair comparison, the ROIs were chosen at the same position in the imaging FOV among the B-mode images obtained at different time points and at different imaging frequencies.

To further evaluate the accumulated noise after the first step of signal processing (converting IQ data to axial displacement), the same ROIs were chosen for computing the means and STDs of axial displacement. To examine the time-varying characteristics of the noise, the evaluation process was performed on all the frames obtained over a 16-ms time period.

In addition to the noise generated by the imaging system and during the first step of signal processing, the noise originating from the second step of signal processing (converting the displacement movie to a shear wave speed map) also contributes to the noise present in shear wave speed maps. In this study, the noise in shear wave speed maps will also be evaluated. However, the evaluation will not be a standalone process, as discussed above, but rather part of the image quality assessment process, which will be described in the next section.

### 2.5. Image Quality Assessment

In SWEI, the output image of the target tissue can be a shear wave speed map, a shear wave modulus map, or a Young’s modulus map. Because the latter two types of images can be obtained from the shear wave speed map through simple conversion, the shear wave speed map will be used in this work for image quality assessment. The image quality of the speed maps obtained at the three frequencies investigated in this work (5 MHz, 10 MHz, and 15 MHz) was assessed using four measures: SNR, CNR, spatial resolution, and measurement precision.

#### 2.5.1. Signal-to-Noise Ratio

In a shear wave speed map, the signal is defined as the mean speed of an ROI, and the noise is the STD of the same ROI. So, the SNR can be simply obtained by computing the mean signal intensity over a specific ROI and dividing this by the associated STD. In each shear wave speed map, two ROIs of the same size, which are close to each other, were chosen for evaluating SNR: one from the background of the phantom and the other from the inclusion. For comparison, these two ROIs are in the same position in the shear wave speed maps obtained at different imaging frequencies for the same phantom. However, the size and location of the ROI will vary among the three phantoms, which have different inclusion sizes (4.3 mm, 3 mm, and 2.3 mm).

To compute SNRs, the mean values (${\bar{c}}_{b}$, ${\bar{c}}_{i}$) and STDs ($s_{c_{b}}$, $s_{c_{i}}$) of the background and inclusion of each phantom were first calculated for the ROIs chosen. Based on these measurements, the SNRs (${SNR}_{b}$, ${SNR}_{i}$) of the background and inclusion of the phantom were then calculated using the following equations.

(1) $${SNR}_{b}={\bar{c}}_{b}/s_{c_{b}}$$

(2) $${SNR}_{i}={\bar{c}}_{i}/s_{c_{i}}$$

#### 2.5.2. Contrast-to-Noise Ratio

In SWEI, a high SNR is adequate for imaging homogeneous tissue (e.g., a fibrotic liver), but it is insufficient for identifying local inhomogeneous features of the target tissue (e.g., a tumor in the liver). To identify these local features or objects within an image, they need to have different values from their surrounding areas, and the difference should be larger than the local noise. In other words, high CNR is needed for imaging inhomogeneous tissues. In this study, contrast is defined as the difference between the mean speeds of the inclusion (${\bar{c}}_{i}$) and background (${\bar{c}}_{b}$) of a phantom. Because the noise level of the inclusion (${\bar{c}}_{b}$) is usually different from that of the background of the phantom ($s_{c_{b}}$), two CNRs, one based on the noise level of the background (${CNR}_{b}$) and the other based on the noise level of the inclusion of the phantom (${CNR}_{i}$), will be computed for comparison of the effect of imaging frequency on CNR using the following two equations.

(3) $${CNR}_{b}=({\bar{c}}_{i}-{\bar{c}}_{b})/s_{c_{b}}$$

(4) $${CNR}_{i}=({\bar{c}}_{i}-{\bar{c}}_{b})/s_{c_{i}}$$

#### 2.5.3. Spatial Resolution

Spatial resolution can be thought of as the ability of a medical imaging system to accurately depict two distinct events in space as separate. It can also be thought of as the degree of smearing, or blurring, a medical imaging system introduces to a single event in space [42]. Based on these two perspectives, there are two primary methods to assess spatial resolution. The first method assesses spatial resolution using a tool that contains more than one distinct object or event. A typical example of such a tool is a bar phantom, which contains several groups of parallel lines with different widths. The spatial resolution of an imaging system is characterized by the group of parallel lines with the smallest width that the imaging system can resolve. The second method for assessing spatial resolution uses only one object or event. Theoretically, given the point spread function (PSF) or line spread function (LSF) of an imaging system, the spatial resolution can be quantified by its full width at half maximum (FWHM) [42]. However, it is practically hard to acquire the PSF or LSF of an imaging system. An alternative method to assess the spatial resolution of an imaging system using a single object, or even to use the edge spread function, which is the convolution of the LFS with a step function [43]. Since the sharp change in material, which leads to a rapid variation in shear wave speed, occurs at the boundary between the inclusion and the background of the phantom, edges can be defined and utilized to characterize the spatial resolution.

**Figure 3:**
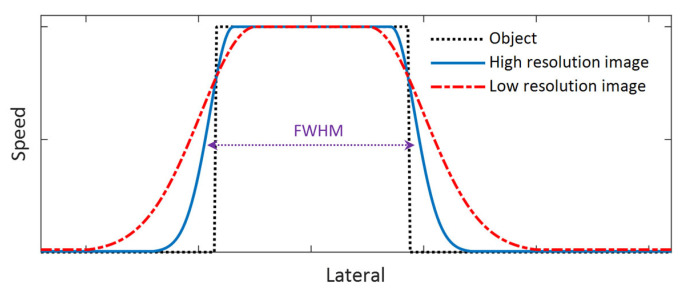
Schematics of an object profile and two example image profiles with different spatial resolution.

Because the primary purpose of this study is to compare the performance of different shear wave tracking frequencies in SWEI, we will not quantify the spatial resolution of the system; instead, we will compare the performance of each imaging frequency in detecting the edge of the inclusion. To do this, we will compare the speed profiles of the shear wave speed map that laterally crosses the center of the inclusion phantom and the spatial resolution will be assessed based on two measures: the slope of the rising and falling sections of the speed profile (corresponding to the edge of the object) and the FWHM of the speed profiles as shown in Figure 3. A larger slope can be observed at the rising and falling sections of the speed profile for an image with higher spatial resolution and vice versa. In addition, the FWHM will be computed for all the phantoms, and it is defined as the lateral width of the inclusion at a height equal to the mean speed of the inclusion and that of the background of the phantom. For this measure, a larger FWHM results from a lower spatial resolution and vice versa.

#### 2.5.4. Measurement Precision

Medical imaging methods/techniques can be generally categorized into two types: qualitative imaging and quantitative imaging. Qualitative imaging, to which most current imaging techniques belong, can provide anatomical and/or functional information about target tissues; however, the image intensity is not a meaningful physical property of the target tissue. Quantitative imaging, however, can provide quantitative evaluation of a specific property of the target tissue, in addition to anatomical and/or functional information. Elasticity imaging, for example, images tissue by evaluating its elasticity (either shear modulus or Young’s modulus) at each pixel.

Like any quantitative measurement technique, measurement accuracy and precision are essential criteria for assessing the effectiveness of SWEI. Accuracy is a measure of the difference between the actual value of a quantity and its measurements. This difference is typically evaluated by error, and a larger error indicates lower accuracy, and vice versa. Because the accuracy of SWEI has been assessed using a widely accepted and reliable method for measuring material elasticity, such as mechanical compression testing (the gold standard method) [44,45], it will not be the focus of this work. Precision is a measure of the difference between different measurements of the same quantity, and it is an essential indicator of the reproducibility of an imaging method. In this work, the shear wave speeds in the background and the inclusion of a phantom were measured three times at each imaging frequency. These measurements will be used to qualitatively assess the effect of imaging frequency on measurement precision.

## 3. Results and Discussion

### 3.1. Comparison of the System Noise Levels at Different Imaging Frequencies

**Figure 4:**
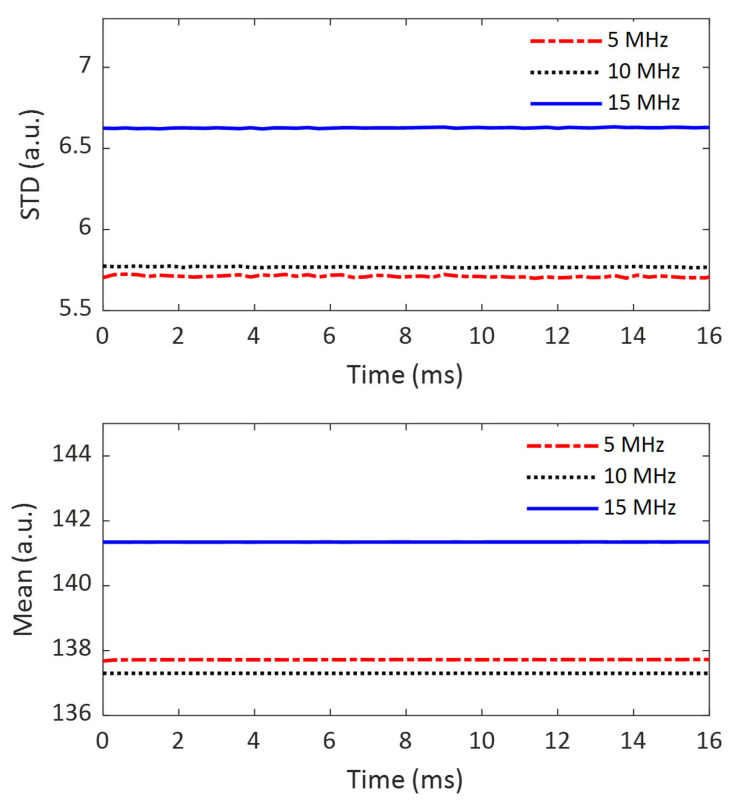
Comparison of the standard deviation (the top subfigure) and mean (the bottom subfigure) of the imaging system at 3 different frequencies over a time period of 16 ms.

In this study, the imaging system refers to imaging transducers and the ultrasound system, as shown in Figure 1. The noise level of this imaging system was evaluated by the STD of a 10 mm-by-10 mm ROI in the imaging FOV. The system noise levels at three imaging frequencies (5 MHz, 10 MHz, and 15 MHz) are compared in the upper figure of Figure 4. For reference, the mean values of the ROI are also displayed in Figure 4.

From the top subfigure of Figure 4, the following observations can be made. First, the system noise obtained at all three frequencies remains nearly constant over time, which is expected for a stable imaging system. Second, the system noise level increases as imaging frequency increases: the noise level at 5 MHz is the smallest, and that at 15 MHz is the largest. The difference between the noise levels at 5 MHz and 10 MHz is only about 1%, but the difference between 5 MHz and 15 MHz is as significant as 16%. Considering the fact that imaging at these three frequencies was performed using two different transducers (5 MHz and 10 MHz using L11-4v; 15MHz using L22-14vLF), it is safe to conclude that the significant difference in system noise level between the lower imaging frequencies (5 MHz and 10 MHz) and the higher frequency (15 MHz) results from the difference in imaging transducers. The imaging transducer with a higher frequency seems to generate more system noise.

Like the system noise levels, the mean values of the ROI are also consistent over time and fall into two groups associated with two different imaging transducers. The only difference is that the mean value at 5 MHz is slightly smaller than that at 10 MHz.

### 3.2. Comparison of the Accumulated Noise Levels in Displacement Detection at Different Imaging Frequencies

The noise in displacement maps originates from two sources: the imaging system and the signal processing method used to extract axial displacement from experimentally recorded IQ data. With knowledge of the system noise levels at three imaging frequencies, it is interesting to see how much noise the signal processing process can generate.

**Figure 5:**
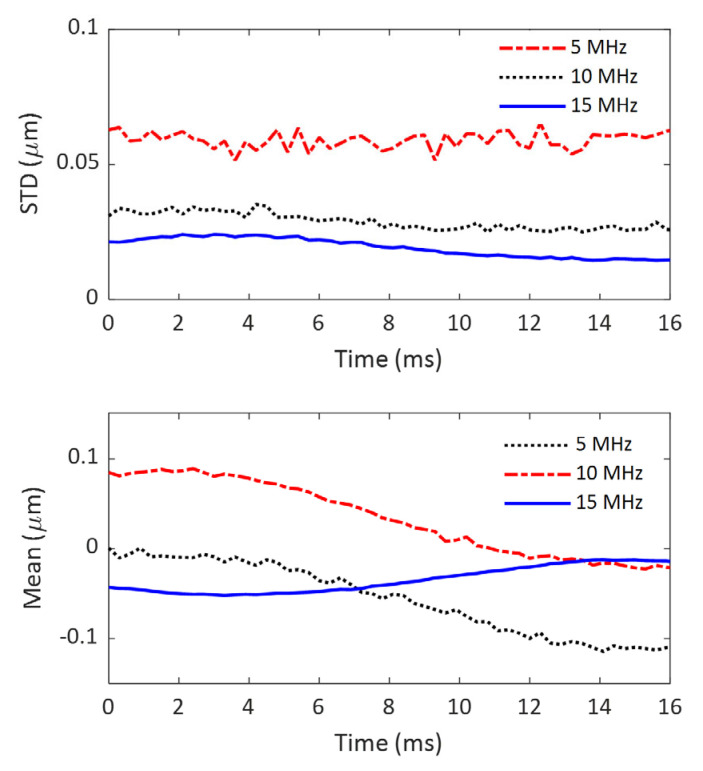
Comparison of the standard deviation (the top figure) and mean (the bottom figure) of the axial displacement evaluation at 3 different frequencies over a time period of 16 ms.

The noise levels (also represented by STD), as well as the mean values computed from the same ROIs as used in evaluating system noise levels, are compared in Figure 5. Similarly to the system noise levels observed in Figure 4, the noise level measurements at each imaging frequency are relatively consistent over time, indicating that the signal processing process did not introduce temporal instability into the displacement evaluation. But, opposite to the observation of system noise levels, the noise in axial displacement decreases as the imaging frequency increases. The imaging at 15 MHz yields the smallest noise values, which are approximately 28% smaller than those obtained at 10 MHz and about 67% smaller than those obtained at 5 MHz. Since shear waves are generated under the same conditions in this study and therefore have the same strength at the exact location of their propagation path, this observation suggests that a higher imaging frequency will lead to a higher SNR (due to a lower noise level) in the entire imaging FOV.

It is necessary to report that, different from the mean values of system noise observed in Figure 5, the mean values of axial displacement vary over time for all three cases. Interestingly, the time-variation patterns for 5 MHz and 10 MHz are almost identical, which is probably because the same transducer produces them.

### 3.3. The Effect of Shear Wave Decay on Shear Wave Speed Evaluation

**Figure 6:**
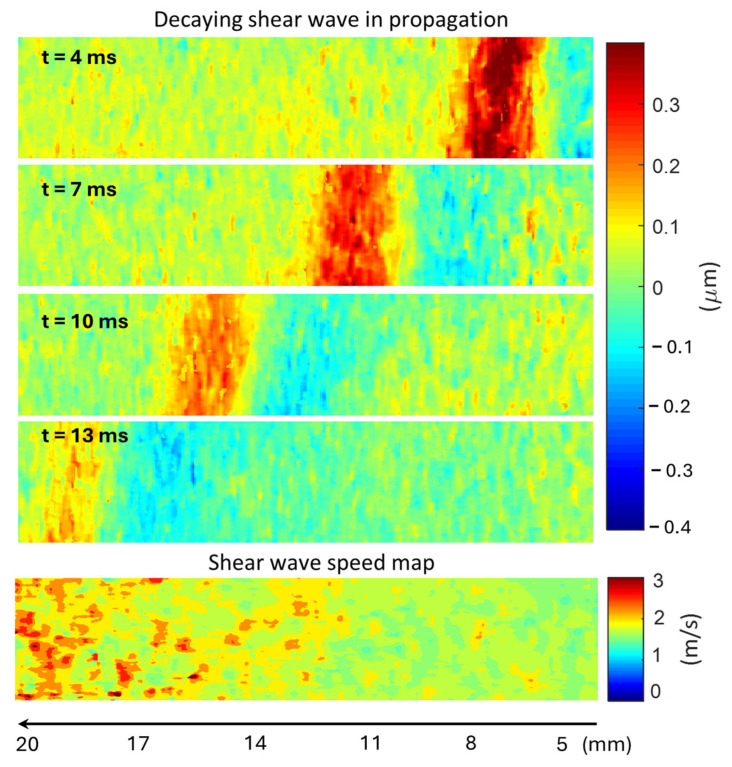
The effect of shear wave decay on shear wave speed evaluation along the wave propagation path. The source of shear wave excitation is on the right-hand side (0 mm), and the shear wave propagates to the left.

Acoustic waves decay during propagation in any medium due to energy spreading and attenuation (scattering and absorption). In SWEI, the amplitude of the generated shear waves will decrease along the propagation pathway. With the assumption that the noise level in displacement evaluation is relatively uniform throughout the imaging FOV, the amplitude decay of the shear wave will result in a decreasing SNR along the shear wave propagation path. The effect of such shear wave decay on shear wave speed evaluation is demonstrated in Figure 6.

In Figure 6, the first four subfigures are the snapshots of a shear wave propagating in the homogeneous gelatin phantom from right to left with 3 3-ms intervals. It can be readily seen that the shear wave amplitude drops along the path of propagation, and so does the shear wave SNR. The consequence of this decay in shear wave speed evaluation (as well as tissue elasticity evaluation) is shown in the last sub-figure: as the shear wave decays (from right to left), increased noise/artifact is generated in the speed map. In other words, the shear wave speed noise is highly correlated to the shear wave displacement SNR.

To reduce the noise level in shear wave speed evaluation, two strategies can be adopted. The first strategy is to increase the strength of the generated shear wave. The parameters of the shear wave excitation sub-system in Figure 1 can be optimized to create a stronger ARFI. However, this strategy is limited by the biosafety requirement, and the strength of ARFI cannot exceed the limit that could cause damage to the target tissue. The second strategy is to reduce the noise level in displacement evaluation, which is the primary goal of this study. Through the comparison of displacement noise at different frequencies shown in Figure 5, it is very promising that high-frequency shear wave tracking will lead to a low-noise shear wave speed map. The effect of the imaging frequency on the image quality in SWEI will be discussed in the following sections.

### 3.4. The Effect of Imaging Frequency on the SNRs of Shear Wave Speed Evaluation

To assess the shear wave tracking frequency on the image quality of SWEI, we first evaluated the SNRs of the speed maps of the three phantoms tested in the study and then compared these SNRs.

#### 3.4.1. SNR Evaluation for the Phantom with a 4.3 mm Inclusion

**Figure 7:**
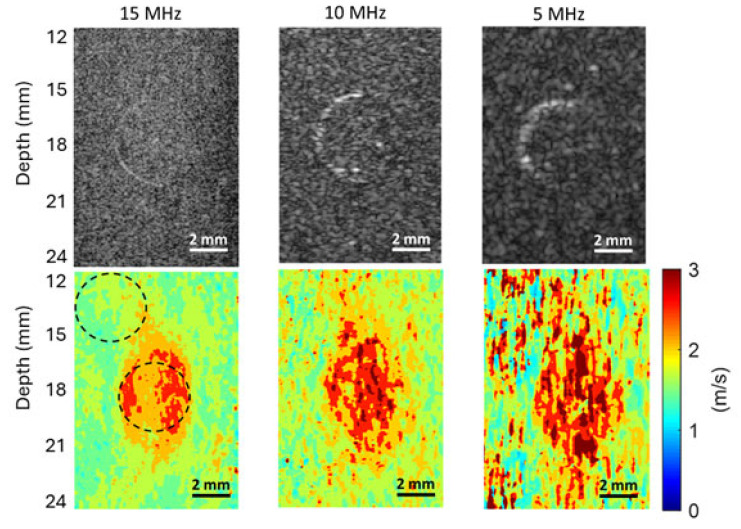
Comparison of the B-mode images (the top row) and shear wave speed maps (the bottom row) of the phantom with a 4.3 mm inclusion obtained at different imaging frequencies. A 2 mm scale bar is included in each sub-figure.

The B-mode images and shear wave speed maps of the phantom with a 4.3 mm inclusion, obtained at frequencies of 5 MHz, 10 MHz, and 15 MHz, are compared in Figure 7. To evaluate the SNRs of the background and inclusion of this phantom, two circular ROIs of the same size were chosen, as depicted in the shear wave speed map obtained at 15 MHz. Such ROIs were also defined on the shear wave speed maps obtained at 10 MHz and 5 MHz, and they have the same size and location as those on the 15 MHz speed map.

The mean values (${\bar{c}}_{b}$, ${\bar{c}}_{i}$) and STDs ($s_{c_{b}}$, $s_{c_{i}}$) of the background and inclusion of this phantom computed from the chosen ROIs were listed in Table 1. Based on these measurements, the SNRs (${SNR}_{b}$, ${SNR}_{i}$) of the background and inclusion of this phantom were also computed using Equations (1) and (2), respectively.

It can be readily seen from Figure 7 that as the imaging frequency decreases, the noise level of the shear wave speed map increases dramatically. Accordingly, the SRNs for both the background and inclusion of the phantom significantly increase with the increasing frequency, as shown in Table 1.

#### 3.4.2. SNR Evaluation for the Phantom with a 3 mm Inclusion

**Figure 8:**
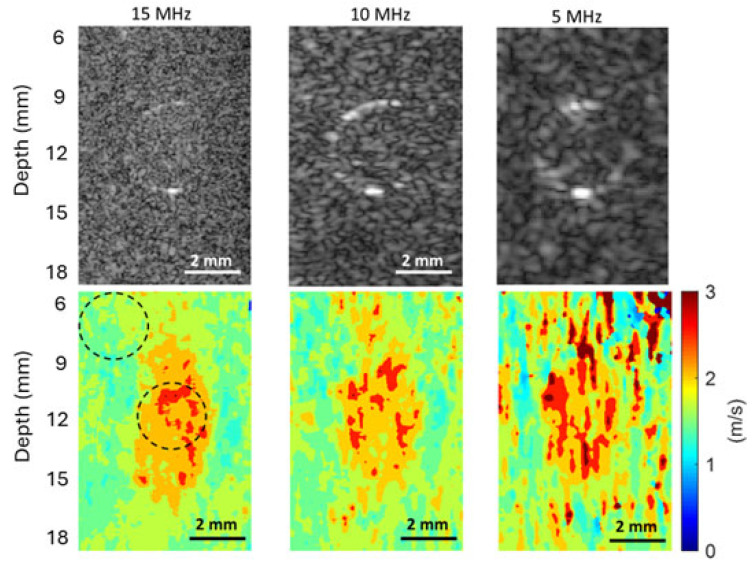
Comparison of the B-mode images (the top row) and shear wave speed maps (the bottom row) of the phantom with a 3 mm inclusion obtained at different imaging frequencies. A 2 mm scale bar is included in each sub-figure.

Similarly to the phantom with a 4.3 mm inclusion, the B-mode images and shear wave speed maps of the phantom with a 3 mm inclusion obtained at 5 MHz, 10 MHz, and 15 MHz were compared in Figure 8. To evaluate the SNRs of the background and inclusion of this phantom, two circular ROIs of the same size were also chosen for all three cases, as depicted in the shear wave speed map obtained at 15 MHz. The mean values, STD, and the SNRs of the background and inclusion of this phantom were also computed from the chosen ROIs and listed in Table 2. From both Figure 8 and Table 2, it can be concluded that a higher imaging frequency leads to a lower noise level in speed maps and, therefore, a higher SNR.

#### 3.4.3. SNR Evaluation for the Phantom with a 2.3 mm Inclusion

**Figure 9:**
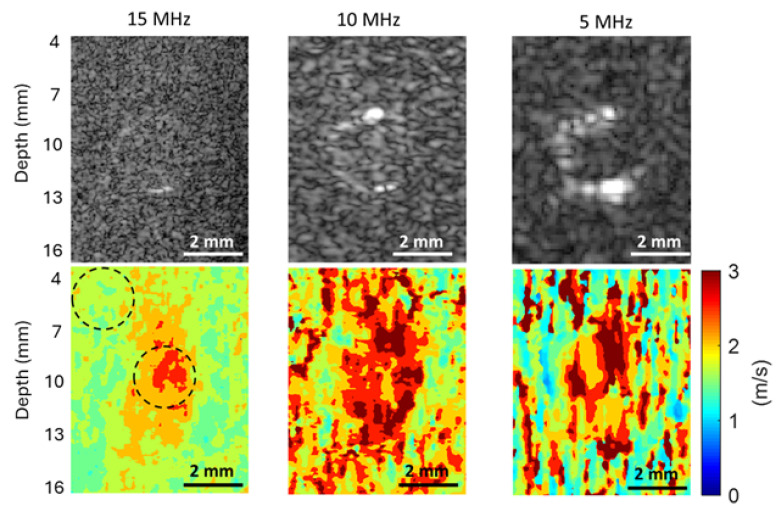
Comparison of the B-mode images (the top row) and shear wave speed maps (the bottom row) of the phantom with a 2.3 mm inclusion obtained at different imaging frequencies. A 2 mm scale bar is included in each sub-figure.

For the phantom with a 2.3 mm inclusion, the B-mode images and shear wave speed maps obtained at 5 MHz, 10 MHz, and 15 MHz were compared in Figure 9. Two circular ROIs of the same size were also chosen for all three cases, as depicted in the shear wave speed map obtained at 15 MHz for computing the SNRs of the background and inclusion of this phantom. The mean values, STDs, and the SNRs of the background and inclusion of this phantom are listed in Table 3.

The observation from Figure 9 and Table 3 confirms the observations made from Figure 7 and Table 1 for the phantom with a 4.3 mm inclusion and those from Figure 8 and Table 2 for the phantom with a 3 mm inclusion. These results clearly show that higher imaging frequency can provide higher SNR for shear wave speed evaluation in SWEI.

#### 3.4.4. SNR Comparison Among Different Imaging Frequencies

**Figure 10:**
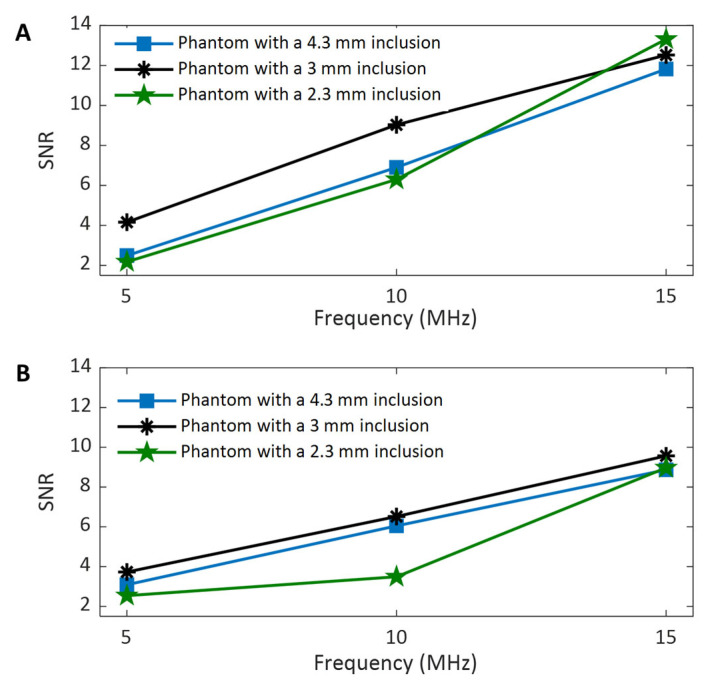
Comparison of the SNRs of the background region (**A**) and inclusion region (**B**) of the shear wave speed maps obtained at different frequencies.

To assess the effect of imaging frequency on the SNRs in shear wave speed evaluation, the SNRs computed from the background and inclusion regions of all three phantoms were depicted in Figure 10A and Figure 10B, respectively. For all three phantoms, the SNRs of either the background (Figure 10A) or the inclusion (Figure 10B) increase as the imaging frequency increases. This observation aligns well with the noise level in displacement detection shown in Figure 5, which indicates that a higher imaging frequency corresponds to a lower noise level. These observations strongly suggest that high-frequency imaging for shear wave tracking is an effective way to improve SNR in SWEI. Because the imaging at 5 MHz and 10 MHz was performed using the same transducer (L11-4v), but the imaging at 15 MHz was performed using a different transducer (L22-14vLF), this conclusion remains valid despite the variation in imaging transducer selection. In other words, to achieve a higher SNR or obtain smoother shear wave speed maps in SWEI, a higher imaging frequency should be chosen if the bandwidth of the imaging transducer allows.

It can also be observed that for both background (Figure 10A) and inclusion (Figure 10B), the SNR-frequency curves for the phantoms with larger inclusions (3 mm and 4.3 mm) share a similar variation trend (increasing nearly linearly with frequency), but the curve for the phantom with a smaller inclusion (2.3 mm) has relatively large variation. This might result from the fact that the ROIs for the phantoms with larger inclusions have relatively larger areas than those with smaller inclusions, and therefore, the statistics exhibit less variation.

Another interesting observation in comparing Figure 10A,B is that at the same imaging frequency, especially at higher frequencies (10 MHz and 15 MHz), the SNRs of the background of the phantom are larger than their corresponding SNRs of the inclusion of the phantom. For example, for the phantom with a 4.3 mm inclusion imaged at 15 MHz, the SNR of the background (11.82) is approximately 33% larger than that of the inclusion (8.87). Since the background and inclusion of phantom are made of 4 *w*/*v*% and 8 *w*/*v*% gelatin, respectively, and therefore they have different stiffness/elasticity, it is possible that this difference in material stiffness results in their difference in speed SNR. Under the same experimental conditions, softer tissues may have a higher SNR, while stiffer tissues may have a smaller SNR.

#### 3.4.5. The Effect of Imaging Frequency on the CNRs of Shear Wave Speed Evaluation

**Figure 11:**
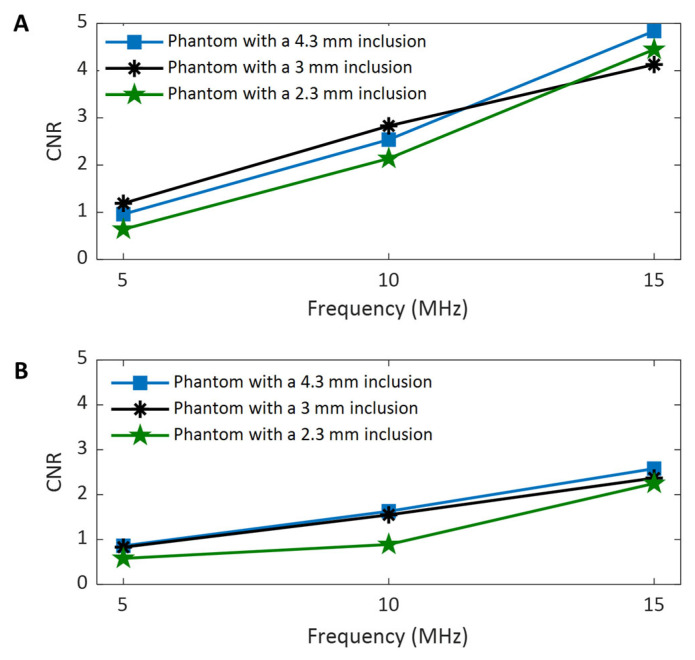
Comparison of the CNRs of the background region (**A**) and inclusion region (**B**) of the shear wave speed maps obtained at different frequencies.

To assess the effect of the imaging frequency on the CNR in SWEI, the two CNRs acquired at different frequencies for all three phantoms are compared in Figure 11A and Figure 11B, respectively. It can be clearly observed from Figure 11 that the values of both CNRs increase as the imaging frequency increases. The higher CNR with increasing frequency makes it easier to identify the phantom inclusions at higher imaging frequencies, as confirmed by the observations in Figure 7, Figure 8 and Figure 9. Because the noise level of the background of phantoms is smaller than that of the inclusion of the phantom, the CNR values in Figure 11A are larger than their counterparts in Figure 11B.

#### 3.4.6. The Effect of Imaging Frequency on the Spatial Resolution of SWEI

To assess the effect of imaging frequency on the spatial resolution of SWEI, we compare here the ability of different imaging frequencies in detecting the edges between the inclusion and background by (i) examining the slope of the speed profile at the edges and (ii) comparing the FWHM values of the three phantoms. To ensure a fair comparison, shear wave speed profiles are to be obtained from Figure 7, Figure 8 and Figure 9 for the phantoms with inclusions of 4.3 mm, 3 mm, and 2.3 mm, respectively. However, the high noise levels in the speed maps obtained at 10 MHz and 5 MHz make it impossible to have a meaningful comparison.

**Figure 12:**
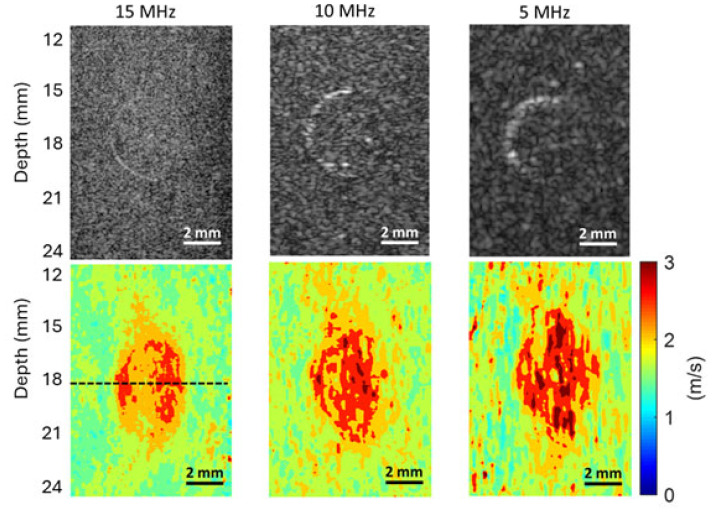
Comparison of the B-mode images (the top row) and shear wave speed maps (the bottom row) of the phantom with a 4.3 mm inclusion obtained at different imaging frequencies. The shear wave speed maps at 10 MHz and 5 MHz are obtained by averaging the displacement data from three sets of data to reduce noise levels. A 2 mm scale bar is included in each sub-figure.

As a compromise, the noise level of the speed maps obtained at 10 MHz and 5 MHz was reduced by averaging three sets of data acquired under the same conditions before extracting the final speed maps. As an example of spatial resolution assessment, this work compares only the results obtained on the phantom with a 4.3 mm inclusion. Figure 12 shows the comparison of the B-mode images obtained at three imaging frequencies and their corresponding shear wave speed maps. It is noted that Figure 12 is identical to Figure 7, except that the speed maps at 10 MHz and 5 MHz are generated based on three sets of experimental data, rather than one set. The shear wave speed profiles were obtained in a lateral position going through the center of the inclusions, as indicated in the speed map at 15 MHz in Figure 12, for all three cases.

**Figure 13:**
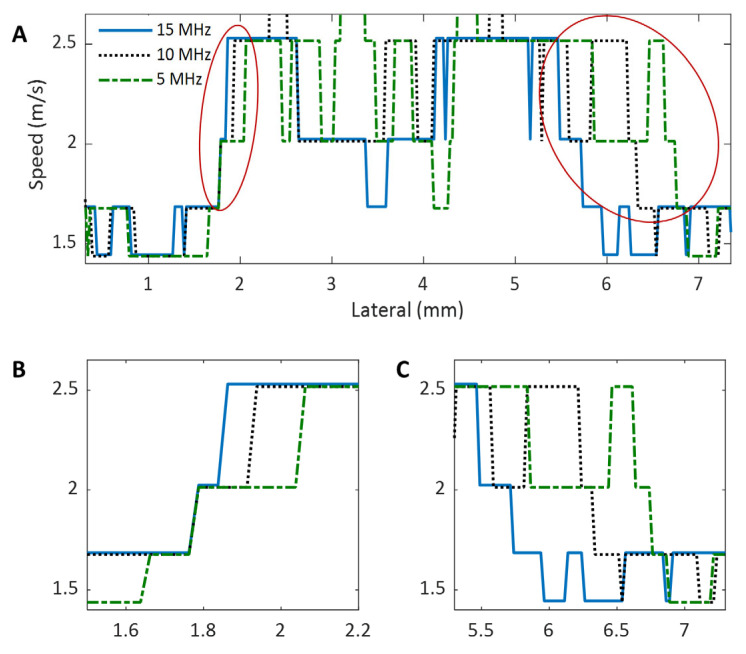
Comparison of the shear wave speed profiles obtained at three different imaging frequencies for the phantom with a 4.3 mm inclusion: (**A**) The entire speed profile. (**B**) The rising part of the speed profiles, as highlighted by the red circle on the left in (**A**). (**C**) The falling part of the speed profiles, as highlighted by the red circle on the right in (**A**).

The shear wave speed profiles obtained at 15 MHz, 10 MHz, and 5 MHz were compared in Figure 13. For clarity, the rising and falling parts of the speed profiles are depicted in Figure 13B and Figure 13C, respectively. To facilitate comparison, we adjusted the location of the speed profiles to ensure the starting positions of their rising parts overlap, as shown in Figure 13B. Although not smooth, it can still be seen from Figure 13B that the 15 MHz speed profile has the steepest rising part, while the 5 MHz profile has the slowest rising part. A similar observation can also be made in Figure 13C.

To compute the FWHMs, we first took the average of the mean speeds of the inclusion and background of the phantom acquired at 10 MHz (2.1 m/s) as the reference (half maximum) and then evaluated the width of the entire speed profiles (as in Figure 13A) at this reference speed. The resultant FWHMs for 15 MHz, 10 MHz, and 5 MHz are 3.7 mm, 4.3 mm, and 4.6 mm, respectively. This result shows that as the imaging frequency decreases, the value of FWHM increases, suggesting that higher shear wave imaging frequencies lead to higher spatial frequencies in SWEI.

#### 3.4.7. The Effect of Imaging Frequency on the Precision of Shear Wave Speed Evaluation

**Figure 14:**
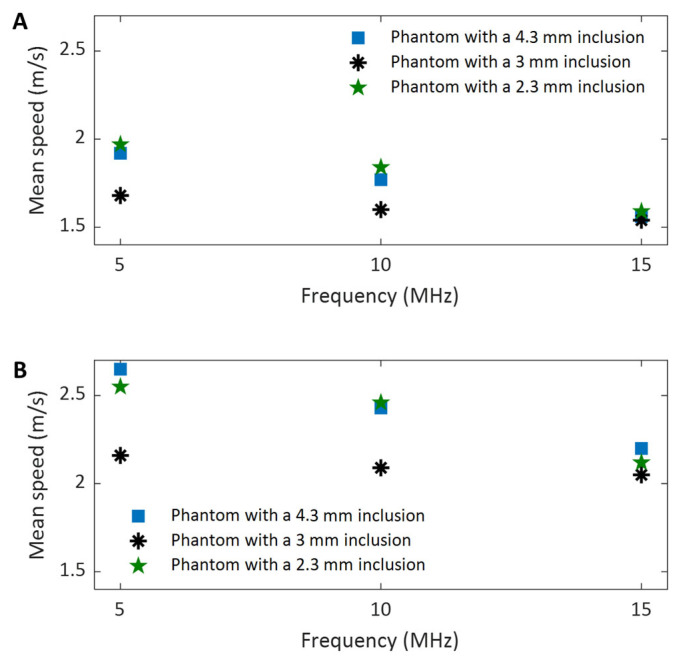
Comparison of the measurement precision of shear wave speed in the (**A**) background region and (**B**) inclusion region of the shear wave speed maps obtained at different frequencies.

To assess the effect of imaging frequency on the precision of shear wave speed measurement, the mean values of shear wave speed calculated for the ROIs in the background, including the three phantoms, are compared in Figure 14A and Figure 14B, respectively. For both cases, it can be observed that as the imaging frequency increases, the data points become increasingly close to each other, resulting in significantly smaller variations among different measurements. This clearly indicates that a higher imaging frequency yields higher precision in evaluating shear wave speed and is therefore preferred in applications.

## 4. Discussion

### 4.1. On the Accuracy of Inclusion Size Measurement in the Depth Direction

From Figure 7, Figure 8 and Figure 9, we can observe that the elasticity images (represented by speed maps of inclusion) of all three inclusions are not circular, as expected, but elongated in the depth (vertical) direction. This phenomenon, that the vertical dimension of elasticity observed is larger than expected, is common in SWEI [46,47,48], and it is an artifact related to the mechanism of this imaging method. Many factors can contribute to this artifact, and the primary causes include the physical process of wave interaction and the digital process of signal processing.

First, the interaction of shear waves and inclusions can cause nonplanar waves near the top and bottom boundaries between the inclusions and the background. In SWEI, when shear waves, which are assumed to be a planar wave in the vertical plane and propagating horizontally, encounter a surface that is neither perpendicular nor parallel to their propagation direction, the wavefront will not be planar anymore. These nonplanar (tilted) waves normally exist near the top and bottom regions of the inclusion. Because SWEI assumes shear waves are planar during the signal processing process to generate the elasticity map, artifacts will occur in the regions of these tilted waves (the top and bottom regions of inclusions), generating vertically elongated inclusion maps.

Second, the horizontal window or kernel used in most SWEI signal processes can also shorten the lateral dimension of inclusions. Again, SWEI assumes that shear waves are planar and propagate horizontally, thus a cornel of certain width is selected in the horizontal direction when extracting shear wave speed. Typically, a larger kernel can provide a smoother elasticity map, but it can also lead to an underestimation of the horizontal dimension of the inclusion. In this study, we believe both physical and digital causes together contribute to the elongated inclusions in the elasticity maps, with the physical cause as the primary cause.

### 4.2. Limitations and Challenges of the Dual-Transducer Approach

In the proposed dual-transducer SWEI, a higher frequency is usually used for tracking shear wave motions. Although high-frequency ultrasound increases the spatial resolution, it also limits the depth of target tissues to be imaged. Typically, high-frequency imaging is used for imaging targets near the interface instead of deep targets. This is also the reason that we generate inclusions of different sizes at different depths of the phantom in this study.

A common challenge of SWEI is the decreasing quality of the elasticity map as the shear wave propagates away from the excitation site. The main cause of this image quality degradation is the decreased SNR of shear waves due to the attenuation during propagation. With the dual-transducer approach, this problem can be remedied using an appropriately selected shear wave generation transducer and related working parameters such as excitation frequency and duration.

As in any other SWEI technique, challenges will undoubtedly occur when applying the dual-transducer method to measure biological tissues. These challenges mainly arise from the complex nature of biological tissues, including their heterogeneity, viscoelasticity, and high attenuation of acoustic waves. Since these challenges are not unique to the dual-transducer approach, we can refer to the literature for potential solutions. Given the flexibility the dual-transducer approach provides, it is highly possible that these challenges can be better addressed compared with the current single-transducer approach.

### 4.3. Considerations for SWEI Approach Selection

Including this work, the literature presents two experimental configurations for SWEI: the single-transducer approach and the dual-transducer approach. In the single-transducer SWEI, shear waves are generated and tracked using the same transducer. Within the frequency coverage of the transducer, proper frequencies should be chosen for shear wave generation and tracking, respectively. Typically, a relatively lower frequency will be used for shear wave generation because low-frequency ultrasound allows for higher power, which will increase the depth of penetration and SNR in the shear waves; a relatively higher frequency will be used for shear wave tracking primarily due to its higher spatial resolution in detecting wave motion. The biggest advantages of this single-transducer approach include its ease of use (only one transducer is needed) and its wide availability (available on most commercial ultrasound machines). So, in practice, the single-transducer approach should be chosen for a defined task as long as the frequency coverage of the transducer satisfies the task requirements. However, if the transducer fails in either shear wave generation or tracking for a defined task, the dual-transducer approach should be adopted. The example scenarios include, but are not limited to, the case where the desired shear wave penetration and the shear wave motion tracking requirements cannot be achieved using a single transducer.

As mentioned earlier in this study, the primary purpose and advantage of the dual-transducer configuration of SWEI is to optimize the performance of both shear wave generation and tracking for a defined application. For example, in imaging small inclusions or target tissues, this approach enables the use of high-frequency ultrasound for tracking shear waves. In the meantime, this approach can also enable high tissue penetration.

### 4.4. The Effect of Shear Wave Tracking Frequency on SWEI Performance

In evaluating the performance of the proposed dual-transducer approach, this study demonstrates that high shear wave tracking frequency outperforms low frequency in SWEI across all four image quality assessment criteria used: SNR (Figure 10), CNR (Figure 11), spatial resolution (Figure 13), and measurement precision (Figure 14). Although it is difficult to provide a definitive explanation without purposefully designed additional research, we believe the most likely reason is the small wavelength and thus high spatial resolution in B-mode imaging that high tracking frequency offers. In SWEI, particle displacements caused by shear wave motion are very small (at the micrometer level, as shown in Figure 6) and are extracted using correlation-based methods from B-mode images obtained with imaging transducers. As shown in Figure 7, Figure 8 and Figure 9, higher frequency B-mode imaging produces smaller speckles, and these smaller speckles can result in more accurate measurements of shear wave particle motions. Because all the signal processing steps leading to an elasticity map, including shear wave speed calculation and elasticity calculation, depend on the extracted particle motions of shear waves, high-accuracy particle motions produced in high-frequency tracking will subsequently lead to improved performance in the elasticity images.

### 4.5. Future Work

As stated in the Introduction of this work, the performance of SWEI is determined by two main factors: the effectiveness of shear wave excitation and the quality of shear wave tracking/recording. In this study, we demonstrated the role of imaging frequency in the performance of SWEI, but we did not investigate the role of shear wave excitation. In future work, the effects of shear wave excitation parameters, such as duration, amplitude, and beam width of ARFI, on the quality of shear wave speed maps should be investigated. Such an investigation could provide guidelines for choosing optimal shear wave excitation parameters for specific applications. Together with the results presented in this work, the knowledge of how to choose optimal excitation parameters for a defined application will lead to a custom-designed SWEI, which can produce the best possible results.

## 5. Conclusions

To meet the need for characterizing the elasticity of small biological tissues or organs, or for identifying small tissue structures within surrounding tissue with different stiffness, we proposed and assessed a dual-transducer approach for high-frequency SWEI in this study. In this approach, two ultrasound transducers and, accordingly, two subsystems are proposed for shear wave generation and tracking, respectively, as opposed to a single transducer in conventional SWEI. This proposal is to address the limitations of the conventional SWEI, where a single transducer is used for both shear wave generation and tracking, but the limited bandwidth of a single transducer does not allow for simultaneous high-frequency imaging and low-frequency excitation that are required for specific applications such as elasticity imaging for lymph nodes, coronary arteries, and tissues of interest in small animals. Then, we experimentally demonstrated the effectiveness of the proposed dual-transducer approach by investigating the effect of shear wave tracking frequency on the performance of SWEI. By comparing the SNR, CNR, spatial resolution, and measurement precision of the results obtained at three different frequencies, we demonstrated that higher-frequency shear wave tracking can achieve higher SNR, CNR, spatial resolution, and measurement precision in SWEI.

## Figures and Tables

**Table 1:** Statistics of shear wave speed in the background and inclusion for the phantom with a 4.3 mm inclusion.

*f* (MHz)	${\bar{c}}_{b}$(m/s)	$s_{c_{b}}$(m/s)	${SNR}_{b}$	${\bar{c}}_{i}$(m/s)	$s_{c_{i}}$(m/s)	${SNR}_{i}$	${CNR}_{b}$	${CNR}_{i}$
5	1.97	0.77	2.49	2.65	0.86	3.09	0.96	0.86
10	1.77	0.26	6.90	2.43	0.40	6.05	2.54	1.63
15	1.56	0.13	11.82	2.20	0.25	8.87	4.84	2.58

**Table 2:** Statistics of shear wave speed in the background and inclusion for the phantom with a 3 mm inclusion.

*f* (MHz)	${\bar{c}}_{b}$ (m/s)	$s_{c_{b}}$ (m/s)	${SNR}_{b}$	${\bar{c}}_{i}$ (m/s)	$s_{c_{i}}$ (m/s)	${SNR}_{i}$	${CNR}_{b}$	${CNR}_{i}$
5	1.68	0.40	4.16	2.16	0.58	3.73	1.19	0.83
10	1.60	0.18	9.03	2.09	0.32	6.52	2.83	1.55
15	1.54	0.12	12.52	2.05	0.21	9.57	4.13	2.37

**Table 3:** Statistics of shear wave speed in the background and inclusion for the phantom with a 2.3 mm inclusion.

*f* (MHz)	${\bar{c}}_{b}$ (m/s)	$s_{c_{b}}$ (m/s)	${SNR}_{b}$	${\bar{c}}_{i}$ (m/s)	$s_{c_{i}}$ (m/s)	${SNR}_{i}$	${CNR}_{b}$	${CNR}_{i}$
5	1.97	0.90	2.18	2.55	1.01	2.54	0.64	0.58
10	1.84	0.29	6.30	2.46	0.70	3.49	2.14	0.89
15	1.59	0.12	13.31	2.12	0.24	8.97	4.45	2.25

## Data Availability

The data are available upon request to the first author.

## Abbreviations

The following abbreviations are used in this manuscript:

SWEI	Shear wave elasticity imaging
ARFI	Acoustic radiation force impulse
IQ	In-phase quadrature
SNR	Signal-to-noise ratio
CNR	Contrast-to-noise ratio
ROI	Region of interest
STD	Standard deviation
PSF	Point spread function
LSF	Line spread function
FWHM	Full width at half maximum

## References

[1] Manduca A., Dutt V., Borup D.T., Muthupillai R., Ehman R.L., Greenleaf J.F. Reconstruction of elasticity and attenuation maps in shear wave imaging: An inverse approach. Proceedings of the International Conference on Medical Image Computing and Computer-Assisted Intervention.

[2] Macé E., Cohen I., Montaldo G., Miles R., Fink M., Tanter M. (2011). In Vivo Mapping of Brain Elasticity in Small Animals Using Shear Wave Imaging. IEEE T. Med. Imaging.

[3] Nightingale K., Palmeri M., Rouze N., Rosenzweig S., Wang M., Trahey G. (2011). Quantitative Elasticity Imaging with Acoustic Radiation Force Induced Shear Waves. Med. Phys..

[4] Bhatia K.S.S., Cho C.C.M., Tong C.S.L., Yuen E.H.Y., Ahuja A.T. (2012). Shear Wave Elasticity Imaging of Cervical Lymph Nodes. Ultrasound. Med. Biol..

[5] Li W.J., Wei Z.T., Yan R.L., Zhang Y.L. (2012). Detection of placenta elasticity modulus by quantitative real-time shear wave imaging. Clin. Exp. Obstet. Gyn..

[6] Tanter M., Fink M. (2014). Ultrafast Imaging in Biomedical Ultrasound. IEEE Trans. Ultrason. Ferr. Freq. Control.

[7] Thiele M., Detlefsen S., Moller L., Madsen B., Hansen J., Fialla A., Trebicka J., Krag A. (2016). Transient and 2-Dimensional Shear-Wave Elastography Provide Comparable Assessment of Alcoholic Liver Fibrosis and Cirrhosis. Gastroenterology.

[8] Herrmann E., de Lédinghen V., Cassinotto C., Chu W., Leung V., Ferraioli G., Filice C., Castera L., Vilgrain V., Ronot M. (2018). Assessment of biopsy-proven liver fibrosis by two-dimensional shear wave elastography: An individual patient data-based meta-analysis. Hepatology.

[9] Isa H.M., Abdulla M., Abdulla M., Al-Hashimi H., Dunne K., Blackwell J. (2025). Utilization of Shear Wave Elastography in the Evaluation of Pediatric Liver Transplant: A Prospective Case-Controlled Study. Pediatr. Transplant..

[10] Charoenchue P., Khorana J., Chitapanarux T., Inmutto N., Chiangmai W.N., Amantakul A., Pojchamarnwiputh S., Tantraworasin A. (2024). Two-Dimensional Shear-Wave Elastography: Accuracy in Liver Fibrosis Staging Using Magnetic Resonance Elastography as the Reference Standard. Diagnostics.

[11] Cosgrove D., Berg W., Doré C., Skyba D., Henry J., Gay J., Cohen-Bacrie C. (2012). Shear wave elastography for breast masses is highly reproducible. Eur. Radiol..

[12] Wang G., Ouyang Y.M., Ruan L.T. (2025). Clinical value of quantitative analysis of Sonazoid-contrast enhanced ultrasound combined with shear wave elastography in discriminating and diagnosing breast tumor characteristics. Front. Oncol..

[13] Qi L.X., Zhou X., Fu Y.G., Zhou W.Y. (2025). Diagnostic value of mammography combined with ultrasound shear wave elastography and magnetic resonance imaging in breast cancer. Oncol. Lett..

[14] Magri F., Chytiris S., Capelli V., Alessi S., Nalon E., Rotondi M., Cassibba S., Calliada F., Chiovato L. (2012). Shear wave elastography in the diagnosis of thyroid nodules: Feasibility in the case of coexistent chronic autoimmune Hashimoto’s thyroiditis. Clin. Endocrinol..

[15] Zhang H.P., Chen M.L., Zou J., Zhou Y.Q. (2025). Value of two-dimensional shear wave elastography quantitative analysis for evaluation of thyroid function in first trimester pregnancy. World J. Radiol..

[16] Abdallah A.A., Mahmoud M.Z., Hassouna M.S., Elkaffas K., Eltelety A.M., Zamzam S.M. (2025). The added value of shear wave elastography when combined to conventional ultrasonography and fine-needle aspiration cytology in detection of malignant thyroid nodule. Egypt. J. Otolaryngol..

[17] Payne C., Watt P., Cercignani M., Webborn N. (2018). Reproducibility of shear wave elastography measuresof the Achilles tendon. Skeletal Radiol..

[18] Yang W.X., Yu Q.R., Wang Y.H., Li D., Tang X.T., Liu W.Y. (2025). The Clinical Value of Real-Time Shear Wave Elastography in Evaluating Closed Complete Achilles Tendon Ruptures. J. Clin. Ultrasound.

[19] Kotha S.R., Blank J.L., Kahr S.M., Reiter A.J., Candan S., Franck C., Thelen D.G. (2025). Shear wave propagation as a noninvasive metric of loading and microdamage in tendon fascicles. J. Mech. Behav. Biomed..

[20] Grn E., Aksakal M., Akdulum I. (2021). Measuring stiffness of normal medial collateral ligament in healthy volunteers via shear wave elastography. Surg. Radiol. Anat..

[21] Zhang H., Elfar J.C., Kwoh C.K., Li Z.M. (2024). Shear wave elastography of transverse carpal ligament increased with simulated carpal tunnel pressure. J. Orthop. Surg. Res..

[22] Wei Y., Alzouhayli K., Schilaty N.D., Hooke A.W., Bates H.A. (2024). Viability of shear wave elastography to predict mechanical/ultimate failure in the anterolateral and medial collateral ligaments of the knee. J. Biomech..

[23] Alfuraih A., O’Connor P., Hensor E., Tan A., Emery P., Wakefield R. (2018). The effect of unit, depth, and probe load on the reliability of muscle shear wave elastography: Variables affecting reliability of SWE. J. Clin. Ultrasound.

[24] Nakao G., Kodesho T., Yamagata K., Adachi R., Ishiyama K., Kozawa K., Watanabe K., Ohsaki Y., Katayose M., Taniguchi K. (2025). Region-specific assessment of the mechanical properties of each hamstring muscle in human cadavers using shear wave elastography. Clin. Biomech..

[25] Haueise A., Carvalho G.F., Azan M., Gehring D., Skerl K., Dieterich A.V. (2025). Development and validation of a semi-automated algorithm to analyze shear wave elastography clips in muscle tissue. Sci. Rep..

[26] Guo L., Zhang Y.P., Li J.D., Ran L., Zhou X., Gao Y., Wu X.X., Li J. (2025). Application Value and Clinical Correlation of Ultrasonic Shear Wave Elastography in Chronic Kidney Disease. Echocardiography.

[27] Abdelgawad A., Abdallah R., Nabil N. (2025). Diagnostic accuracy of shear wave elastography in evaluating renal fibrosis in children with chronic kidney disease: A comparative study with Tc-99 DMSA renal scan. Egypt. J. Radiol. Nuc. Med..

[28] Liu D.L., Lin W.L., Wei C.L., Huang Y.Q., Chen F.J., Zhang Y.L., Pu F.F. (2025). Exploring the potential clinical value of shear wave elastography in prostate cancer: A meta-analysis. BMC Cancer.

[29] Liu X., Zhu J., Shi M.Q., Pan Y.S., Cao X.Y., Zhang Z.X. (2024). Predicting clinically significant prostate cancer in elderly patients: A nomogram approach with shear wave elastography. Prostate.

[30] Al Mutairi F., Alyami J., Aldhebaib A., Wazzan M., Khashoggi K., Abduljabar A., Alotaibi J., Alzahrani T., Alshehri Z., Almosabi S. (2025). Point shear wave elastography application in assessment pancreas tissue stiffness: A pilot study. Radiography.

[31] Cho H., Yang S.W., Suh G.H., Choi J. (2023). Sedative effect with the combination of butorphanol and midazolam on two-dimensional shear wave elastography of pancreas and kidney in healthy dogs. Am. J. Vet. Res..

[32] Civale J., Parasaram V., Bamber J.C., Harris E.J. (2022). High frequency ultrasound vibrational shear wave elastography for preclinical research. Phys. Med. Biol..

[33] Xiao Y., Jin J., Yuan Y., Zhao Y., Li D.D. (2023). Ultrasound shear wave phase velocity imaging using black-box system identification (BSI): A data-driven approach. Phys. Med. Biol..

[34] Nightingale K., McAleavey S., Trahey G. (2003). Shear-wave generation using acoustic radiation force: In vivo and ex vivo results. Ultrasound Med. Biol..

[35] Nightingale K. (2011). Acoustic Radiation Force Impulse (ARFI) Imaging: A Review. Curr. Med. Imaging.

[36] Liu J.F., Foiret J., Stephens D.N., Le Baron O., Ferrara K.W. (2016). Development of a spherically focused phased array transducer for ultrasonic image-guided hyperthermia. Phys. Med. Biol..

[37] Ebbini E., Ter Haar G. (2015). Ultrasound-guided therapeutic focused ultrasound: Current status and future directions. Int. J. Hyperther..

[38] Konofagou E.E., Maleke C., Vappou J. (2012). Harmonic Motion Imaging (HMI) for Tumor Imaging and Treatment Monitoring. Curr. Med. Imaging Rev..

[39] Hashemi H.S., Mohammed S.K., Zeng Q., Azar R.Z., Rohling R.N., Salcudean S.E. (2023). 3-D Ultrafast Shear Wave Absolute Vibro-Elastography Using a Matrix Array Transducer. IEEE Trans. Ultrason. Ferroelectr. Freq. Control.

[40] Yang H., Carrascal C.A., Xie H., Shamdasani V., Anthony B.W. (2020). 2-D Ultrasound Shear Wave Elastography with Multi-Sphere-Source External Mechanical Vibration: Preliminary Phantom Results. Ultrasound Med. Biol..

[41] Evans A., Whelehan P., Thomson K., McLean D., Brauer K., Purdie C., Jordan L., Baker L., Thompson A. (2010). Quantitative shear wave ultrasound elastography: Initial experience in solid breast masses. Breast Cancer Res..

[42] Prince J.L., Links J.M. (2006). Medical Imaging Signals and Systems.

[43] Webb A. (2022). Introduction to Biomedical Imaging.

[44] Liu Y., Liu J.F., Fite B.Z., Foiret J., Ilovitsh A., Leach J.K., Dumont E., Caskey C.F., Ferrara K.W. (2017). Supersonic transient magnetic resonance elastography for quantitative assessment of tissue elasticity. Phys. Med. Biol..

[45] Liu Y., Liu J.F., Fite B.Z., Foiret J., Leach J.K., Ferrara K.W. Quantitative MR-guided transient shear wave imaging for tissue elasticity assessment. Proceedings of the 2016 IEEE International Ultrasonics Symposium (IUS).

[46] Kijanka P., Urban M.W. (2019). Local Phase Velocity Based Imaging: A New Technique Used for Ultrasound Shear Wave Elastography. IEEE T. Med. Imaging.

[47] Cacko D., Lewandowski M. (2022). Shear Wave Elastography Implementation on a Portable Research Ultrasound System: Initial Results. Appl. Sci..

[48] Xiao Y., Jin J., Yuan Y., Zhao Y., Li D.D. (2023). A New Estimation Scheme for Improving the Performance of Shear Wave Elasticity Imaging. Ultrasound Med. Biol..

